# Crystal structures of binuclear complexes of gadolinium(III) and dysprosium(III) with oxalate bridges and chelating *N*,*N*′-bis­(2-oxido­benz­yl)-*N*,*N*′-bis­(pyridin-2-ylmeth­yl)ethyl­enedi­amine (bbpen^2−^)

**DOI:** 10.1107/S2056989019002998

**Published:** 2019-03-05

**Authors:** Guilherme Augusto Barbosa, Francielli Sousa Santana, Giovana Gioppo Nunes, Jaísa Fernandes Soares

**Affiliations:** a Departamento de Química, Universidade Federal do Paraná, Centro, Politécnico, Jardim das Américas, 81530-900, Curitiba-PR, Brazil

**Keywords:** crystal structure, binuclear, lanth­anide, oxalate bridge, *N*,*N*′-bis­(2-hy­droxy­benz­yl)-*N*,*N*′-bis­(pyridin-2-ylmeth­yl)ethyl­enedi­amine, H_2_bbpen

## Abstract

The reaction between mononuclear [*Ln*(bbpen)Cl] [*Ln* = Gd or Dy; H_2_bbpen = *N*,*N*′-bis­(2-hy­droxy­benz­yl)-*N*,*N*′-bis­(pyridin-2-ylmeth­yl)ethyl­enedi­amine, C_28_H_30_N_4_O_2_] and potassium oxalate monohydrate in water/methanol produced the solvated centrosymmetric isostructural binuclear (μ-oxalato)bis­{[*N*,*N*′-bis­(2-oxidobenzyl-κ*O*)-*N*,*N*′-bis­(pyridin-2-ylmethyl-κ*N*)ethyl­enedi­amine-κ^2^ *N*,*N*′]dilanthanide(III)}–methanol–water (1/4/4) complexes, [*Ln*_2_(C_28_H_28_N_4_O_2_)_2_(C_2_O_4_)]·4CH_3_OH·4H_2_O, with lanthanide(III) = gadolinium(III) (*Ln* = Gd) and dysprosium(III) (*Ln* = Dy), in high yields (*ca* 70%) directly from the reaction mixtures. In both complexes, the lanthanide ion is eight-coordinate and adopts a distorted square-anti­prismatic coordination environment. The triclinic (*P* ) unit cell contains one dimeric unit together with four water and four methanol mol­ecules; in the final structural model, two of each type of solvating mol­ecule refine well. In each lanthanide(III) dimeric mol­ecule, the medium-strength O⋯H—O hydrogen-bonding pattern involves four oxygen atoms, two of them from the phenolate groups that are ‘bridged’ by one water and one methanol mol­ecule. These inter­actions seem to contribute to the stabilization of the relatively compact shape of the dimer. Electron densities associated with an additional water and methanol mol­ecule were removed with the SQUEEZE procedure in *PLATON* [Spek (2015 ▸). *Acta Cryst.* C**71**, 9–18]. These two new compounds are of inter­est with respect to magnetic properties.

## Abstract 2

Oxalate-bridged, centrosymmetric binuclear complexes of gadolinium(III) and dysprosium(III) with hexa­dentate bbpen^2–^ (H_2_bbpen = *N*,*N*′-bis­(2-hy­droxy­benz­yl)-*N*,*N*′-bis­(pyridin-2-ylmeth­yl)ethyl­enedi­amine) are isotypic and were synthesized for structural and magnetic susceptibility studies. The dimeric mol­ecules of the complexes crystallize together with water and methanol mol­ecules, with which they form a variety of weak and medium-strength hydrogen bonds.

## Chemical context   

Since the discovery, in 2003, of the first lanthanide(III)-based single-ion magnets (SIM), namely (Bu_4_N)[*Ln*Pc_2_] (H_2_Pc = phthalocyanine; *Ln* = Tb and Dy; Ishikawa *et al.*, 2003 ▸), a number of lanthanide(III) complexes have been prepared for magnetic studies because of their intrinsically high magnetic anisotropy barrier. Heterometallic 3*d–*4*f* single-mol­ecule magnets (SMM) have also been sought, particularly in the early 2000s, mainly because of the possibility of improving magnetic response when compared to *d*-block-only metal complexes such as those of manganese(III), cobalt(II) and nickel(II) (Piquer & Sañudo, 2015 ▸).

Among the 3*d–*4*f* heterometallic systems of higher nuclearity, two tetra­nuclear compounds formulated as [*M*(*μ*-dto)_3_{Dy(HBpz_3_)_2_}_3_]·4CH_3_CN·2CH_2_Cl_2_ (*M* = Fe^III^ or Co^III^; HBpz^−^ = hydro­tris­(pyrazol­yl)borate; dto^2–^ = di­thio­oxalate) presented slow relaxation of the magnetization under applied magnetic field (Xu *et al.*, 2012 ▸). In this three-blade propeller framework, the tris-chelate [*M*(dto)_3_]^3–^ complex forms the central unit, which is bridged to the [Dy(HBpz_3_)_2_]^+^ peripheral positions by the di­thio­oxalate ions. The lanthanide cations assume square-anti­prismatic coordination environments while the *d-*block metal is octa­hedrally coordinated (Xu *et al.*, 2012 ▸). The same monocationic [Dy(HBpz_3_)_2_]^+^ complex had previously been employed to produce binuclear [Dy_2_(*μ*-ox)(HBpz_3_)_4_]·2CH_3_CN·CH_2_Cl_2_, this time with oxalate (ox^2–^) as the bridging ligand. Direct current (DC) magnetic susceptibility measurements performed with this dimeric compound revealed the presence of an intra­molecular ferromagnetic inter­action between the Dy^III^ cations (Xu *et al.*, 2010 ▸). Other oxalate-bridged lanthanide(III) complexes have also shown field-induced slow magnetic relaxation (Zhang *et al.*, 2015 ▸) or weak (anti­ferro)magnetic exchange inter­actions (Feng *et al.*, 2014 ▸). In all cases mentioned above, the products were obtained by self-assembly in one-pot reactions, sometimes under hydro­thermal conditions.

In our research group, we first attempted to prepare heterometallic complexes of general formula [*M* ^III^(*μ*-ox)_3_{*Ln*(bbpen)}_3_] (H_2_bbpen = *N*,*N*′-bis­(2-hy­droxy­benz­yl)-*N*,*N*′-bis­(pyridin-2-ylmeth­yl)ethyl­enedi­amine) *via* modular synthesis employing [*Ln*(bbpen)Cl] (*Ln* ^III^ = Gd or Dy) and K_3_[*M*(ox)_3_] (*M* ^III^ = Cr or Co) as building blocks in a 3:1 proportion. The syntheses with gadolinium(III) and chromium(III) produced colourless crystals of the binuclear complex [{Gd(bbpen)}_2_(*μ*-ox)]·4CH_3_OH·4H_2_O, as revealed by single crystal X-ray diffraction analysis. The formation of this dimer is explained by dissociation of [Cr(ox)_3_]^3–^ into {Cr(ox)_2_(OH_2_)_2_}^−^ and ox^2–^ in aqueous solution (Krishnamurty & Harris, 1960 ▸), followed by inter­action of the ox^2–^ anion with Gd(bbpen)^+^. Structural elucidation of this otherwise unexpected product prompted us to try and perform its targeted preparation with both gadolinium(III) and dysprosium(III) in good yields.
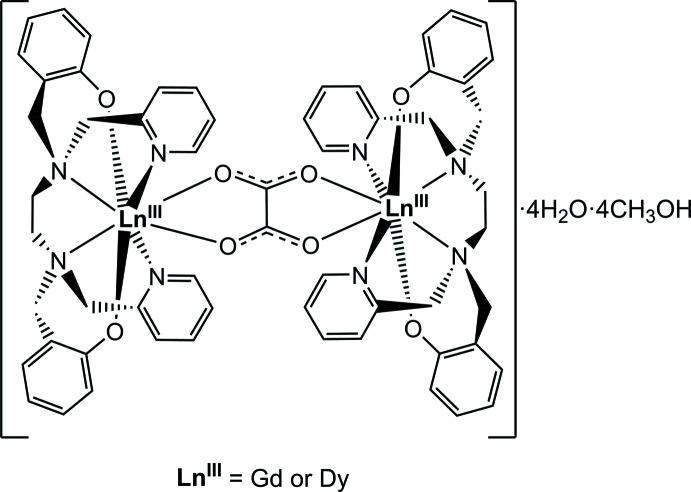


In this paper we report the rational synthesis and the crystal and mol­ecular structures of the two binuclear and solvated [{*Ln*(bbpen)}_2_(*μ*-ox)] products [*Ln* = Gd (**1**) or Dy (**2**)], prepared from the direct reaction between [*Ln*(bbpen)Cl] and K_2_C_2_O_4_·H_2_O in water/methanol media.

## Structural commentary   

**Figure 1:**
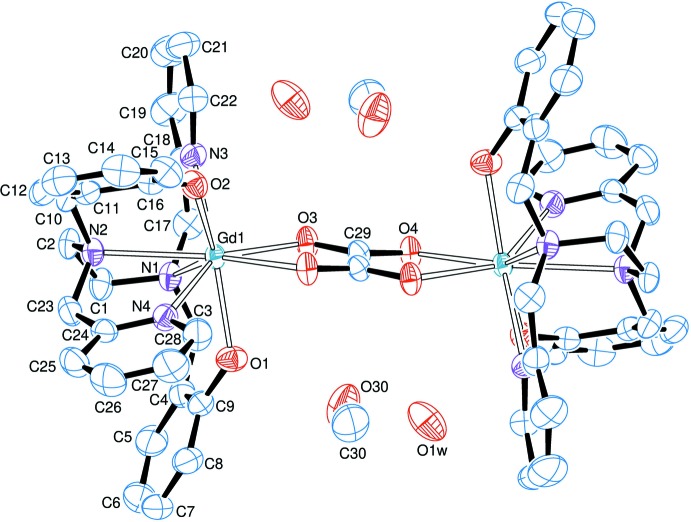
View of [{Gd(bbpen)}_2_(*μ*-ox)]·4CH_3_OH·4H_2_O (compound **1**), with the atom-numbering scheme. Hydrogen atoms have been omitted for clarity. Displacement ellipsoids are drawn at the 50% probability level Unlabelled atoms are generated by the symmetry operation −*x*, −*y* + 1, −*z* + 1.

**Figure 2:**
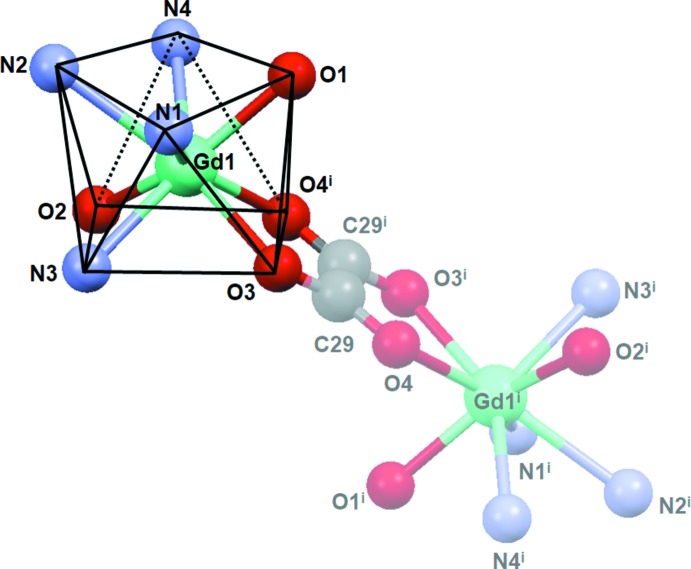
Plot of the coordination sphere about the lanthanide(III) atom in the structure of **1** [symmetry code: (i) −*x*, −*y* + 1, −*z* + 1].

Compounds **1** and **2** are isostructural and crystallize in the *P* 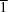 space group, with four methanol and four water mol­ecules per lanthanide dimer. Crystals contain the neutral [*Ln*_2_(*μ*-ox)(bbpen)_2_] mol­ecules (Fig. 1 ▸) in which gadolinium(III) (**1**) or dysprosium(III) (**2**) are eight-coordinate; the [*Ln*(bbpen)]^+^ units are connected to one another by oxalate bridging in the usual bis­(bidentate) coordination mode. The ox^2–^ ligand lies about an inversion centre. The coordination sphere of the lanthanide(III) ion is formed by an N_4_O_2_ donor set from the bbpen^2–^ ligand and two oxygen atoms from the bridging oxalate. In **1** and **2** each metal cation has a distorted square-anti­prismatic coordination environment (Fig. 2 ▸), as indicated by general inspection of atom positions and bond angles, and confirmed from the crystallographic data by the use of the *SHAPE* program (Llunell *et al.*, 2005 ▸). The average *Ln*—N bonds are *ca* 2.60 and 2.58 Å for **1** and **2**, respectively, while the average *Ln*—O distances are *ca* 2.27 (**1**) and 2.24 Å (**2**). The non-bonding Dy⋯Dy distance in **2**, 6.1488 (17) Å, is close to the analogous distance of 6.14 Å in [Dy_2_(*μ*-ox)(HBpz_3_)_4_]·2CH_3_CN·CH_2_Cl_2_ (Xu *et al.*, 2010 ▸). The O3—*Ln*—O4^i^ angles of approximately 68° in both **1** and **2** [symmetry code: (i) −*x*, 1 − *y*, 1 − *z*] are also similar to those reported for the dysprosium(III)–hydro­tris(pirazolylborate) dimer mentioned above. The slightly decreased crystal volume of the Dy compound [1626.3 (7) Å^3^] compared with that of the Gd compound [1633.7 (3) Å^3^] is a perfect match with the smaller effective ionic radius of eight-coordinate Dy^III^ *versus* Gd^III^ (1.027 and 1.053 Å, respectively; Shannon, 1976 ▸), and is in line with the lanthanide contraction. Structural representations provided in this paper are for compound **1**; the dysprosium(III) product **2** gives rise to very similar results.

## Supra­molecular features   

In both structures, the hydrogen atoms from the crystallizing solvents (water and methanol) participate in an extensive three-dimensional hydrogen-bonding network that may be described as medium-strength inter­molecular inter­actions (Tables 1 ▸ and 2 ▸).

**Figure 3:**
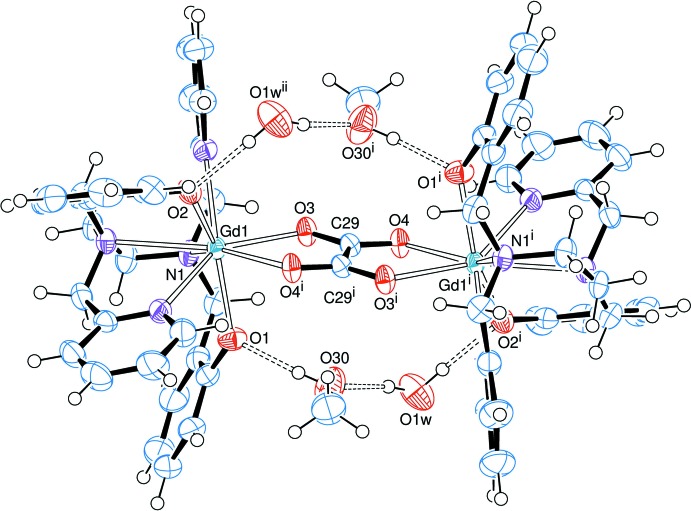
*ORTEP* representation of hydrogen-bonding inter­actions for compound **1** involving solvating methanol and water mol­ecules, with hydrogen bonds indicated by double-dashed lines. Displacement ellipsoid are drawn at the 50% probability level [symmetry codes: (i) −*x*, −*y* + 1, −*z* + 1; (ii) −*x* + 1, −*y* + 1, −*z* + 1.].

**Figure 4:**
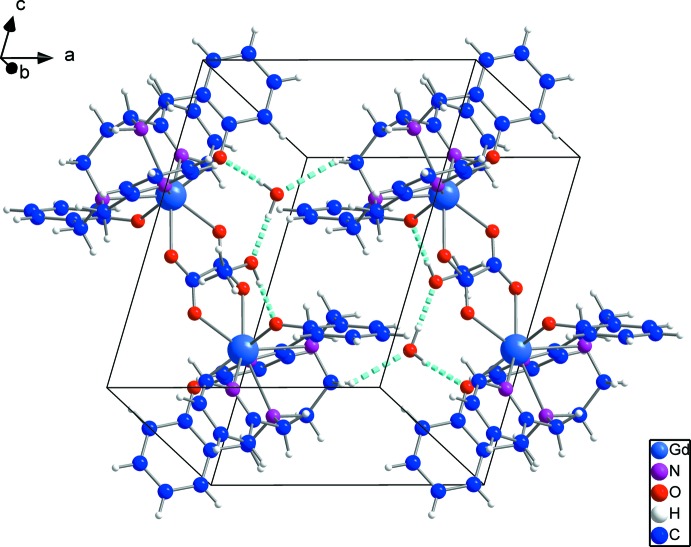
Representation of the dimeric mol­ecules of **1** viewed approximately down the *b* axis of the unit cell. The binuclear complexes are linked through medium-strength hydrogen bonds to solvating water and methanol mol­ecules, and through weak C1—H1*B*⋯O1*W* ^ii^—H⋯O_phenolate_ inter­actions to one another [symmetry code: (ii) −*x* + 1, −*y* + 1, −*z* + 1.].

The solvating (methanol and water) mol­ecules, half of which refine well and are depicted in Fig. 3 ▸, participate in inter­molecular inter­actions with the dimeric complexes **1** and **2**. As seen in Fig. 3 ▸, one water and one methanol mol­ecule are hydrogen-bonded to one another and to the phenolate oxygen atoms in the ligands, generating an O1⋯H—O30⋯H–O1*W*—H⋯O2^iii^ ‘bridge’, as well as a symmetry-related chain on both sides of the plane formed by the metal and oxalate ions. The water mol­ecules in these chains also connect one dimer to another through weak C1—H1*B*⋯O1*W* ^ii^ inter­actions (Fig. 4 ▸; Tables 1 ▸ and 2 ▸).

The other half of the solvent mol­ecules in the unit cell, the electron densities of which have been removed with the SQUEEZE routine in *PLATON* (Spek, 2015 ▸) because of being highly disordered, also contribute to the overall hydrogen-bonding network. This is inferred from the positions of the four main electron-density peaks, which have been assigned to oxygen atoms from the disordered solvents and may give rise to medium-strength to weak hydrogen-bond inter­actions. For **1**, O⋯O distances involving three of these peaks amount to 2.66–2.78 Å as far as O⋯O1*W* contacts are concerned, with O1*W* acting as a potential electron-density acceptor, and are larger than 3.1 Å for O⋯O30 (numbering scheme in Fig. 3 ▸). For **2**, in turn, the corresponding distances are longer than for **1** at 2.88–3.84 Å for O⋯O1*W*, and even larger (> 4.6 Å) for O⋯O30. On the other hand, any possible inter­action involving the phenolate oxygen atoms would be very weak, with the shortest O⋯O contact with the disordered solvents being longer than 4.0 Å.

## Database survey   

Examples of mononuclear lanthanide(III) complexes with bbpen^2–^ and related ligands appear in the literature (Molloy *et al.*, 2017 ▸; Liu *et al.*, 2016 ▸; Yamada *et al.*, 2016 ▸; Gregório *et al.*, 2015 ▸; Qin *et al.*, 2014 ▸; Yamada *et al.*, 2010 ▸; Morss & Rogers, 1997 ▸). Binuclear structures with these hexa­dentate ligands have been reported by Chatterton *et al.* (2005 ▸), and by Setyawati *et al.* (2000 ▸).

## Synthesis and crystallization   

*Ln*Cl_3_·6H_2_O (*Ln* ^III^ = Gd or Dy) and K_2_C_2_O_4_·H_2_O were purchased from Aldrich and used without purification. *N*,*N*′-Bis(2-hy­droxy­benz­yl)-*N*,*N*′-bis­(pyridin-2-ylmeth­yl)ethyl­ene­di­amine (H_2_bbpen) (Neves *et al.*, 1992 ▸) and the [*Ln*(bbpen)Cl] precursors, with *Ln* = Gd or Dy (Liu *et al.*, 2016 ▸), were prepared using adapted procedures described in the literature. Methanol and diethyl ether (Vetec) were used without treatment. Ultrapure water (Milli-Q, Millipore type 1, resistivity of 18.2 MΩ cm at 298 K) was employed as described below.

**Synthesis of [{Gd(bbpen)}_2_(μ–ox)]·4CH_3_OH**·**4H_2_O (compound 1)**

A solution of 8.11 mg (0.0440 mmol) of K_2_C_2_O_4_·H_2_O in 1.0 ml of water was slowly added to a methanol solution of 61.1 mg (0.0947 mmol) of [Gd(bbpen)Cl]. The colourless reaction mixture was stirred at room temperature for *ca* 5 min, and was then cooled down to 277 K to give block-shaped colourless crystals after four days. These were isolated by filtration, washed with diethyl ether and dried. Total yield: 49.0 mg (68.6%) based on the [{Gd(bbpen)}_2_(μ–ox)]·4CH_3_OH·4H_2_O formulation, compound **1**. FTIR (emulsion in mineral oil): 3362, 3198 [*s*, ν(OH)]; 1655 [*s*, ν(CO)_ox_]; 1590, 1568 [*s*, ν(C=N) + ν(C=C)], 1290 [*s*, ν(CO)_phenolate_], 762 and 768 [*m*, δ(C—H)_Ar+py_]. Product **1** is soluble in aceto­nitrile, 1,2-di­meth­oxy­ethane (dme), di­chloro­methane and tetra­hydro­furan. Elemental analysis: calculated for **1** (C_62_H_80_Gd_2_N_8_O_16_) C 49.39, H 5.35, N 7.43%. Found: C 48.56, H 5.49, N 7.45%.

**Synthesis of [{Dy(bbpen)}_2_(μ–ox)]·4CH_3_OH**·**4H_2_O (compound 2)**

A mixture of 61.0 mg (0.0938 mmol) of [Dy(bbpen)Cl] in 9.0 ml of methanol and 8.90 mg (0.0483 mmol) of K_2_C_2_O_4_·H_2_O in 1.0 ml of water was prepared as described for **1**. The resulting solution was cooled at 277 K to produce colourless block-shaped crystals, which were recovered by filtration and washed with diethyl ether. Total yield: 53.9 mg (75.7%) based on the [{Dy(bbpen)}_2_(μ–ox)]·4CH_3_OH·4H_2_O formulation, compound **2**. FTIR (emulsion in mineral oil): 3363, 3198 [*s*, ν(OH)], 1590 [*s*, ν(CO)_ox_]; 1570 (*m*), 1481 (*s*), 1459 [*s*, ν(C=N) + ν(C=C)]; 1290 [*s*, ν(CO)_Ph_], 762 and 768 [*m*, δ(C—H)_Ar+ py_]. The product solubility is similar to that described for **1**. Elemental analysis: calculated for **2** (C_62_H_80_Dy_2_N_8_O_16_) C 49.04, H 5.31, N 7.38%. Found: C 49.02, H 5.71, N 7.56%.

## Refinement   

Crystal data, data collection and structure refinement details for the two structures are summarized in Table 3 ▸. Both **1** and **2** showed high susceptibility to the loss of the crystallization solvent mol­ecules once removed from the mother liquor. Hydrogen atoms in **1** and **2** were included in idealized positions with methyl, methyl­ene and aromatic C—H distances set at 0.98, 0.99 and 0.95 Å, respectively, and O—H at 0.84 Å and refined as riding with *U*_iso_(H) = 1.2–1.5*U*_eq_(C,O). Hydrogen atoms on the water mol­ecules were located in difference-Fourier maps and were refined with distance restraints (DFIX O—H = 0.82 Å for **1** and **2**, DANG = 1.45 Å for **2**).

Both structures present four methanol and four water mol­ecules per unit cell; two of each were treated as diffuse contribution to the overall scattering without specific atom positions and were eventually removed by the use of the SQUEEZE procedure in *PLATON* (Spek, 2015 ▸). The proposed identity of these highly disordered mol­ecules as ‘2H_2_O + 2MeOH’ per unit cell finds support in the total calculated count of 58 and 59 electrons provided by SQUEEZE for **1** and **2**, respectively, as compared with the expected count of 56 electrons. The volume of the void filled by the disordered solvent amounts to 269 and 260 Å^3^ for **1** and **2**, respectively, and corresponds to 16.0–16.5% of the unit cell, in very good agreement with the volume expected for small mol­ecules such as water and methanol. The ratio between the total solvent-accessible void volume and the experimental electron count is of *ca* 4.5 Å^3^ per electron.

## Supplementary Material

Crystal structure: contains datablock(s) 1, 2. DOI: 10.1107/S2056989019002998/wm5489sup1.cif

Structure factors: contains datablock(s) 1. DOI: 10.1107/S2056989019002998/wm54891sup2.hkl

Structure factors: contains datablock(s) 2. DOI: 10.1107/S2056989019002998/wm54892sup3.hkl

CCDC references: 1899963, 1899964

Additional supporting information:  crystallographic information; 3D view; checkCIF report

## Figures and Tables

**Table 1:** Hydrogen-bond geometry (Å, °) for **1** [Chem scheme1]

*D*—H⋯*A*	*D*—H	H⋯*A*	*D*⋯*A*	*D*—H⋯*A*
O30—H30⋯O1	0.84	1.80	2.643 (3)	177
C1—H1*B*⋯O1*W* ^ii^	0.99	2.59	3.459 (3)	147
O1*W*—H1*W*⋯O2^iii^	0.83 (2)	1.96 (2)	2.786 (3)	170 (3)
O1*W*—H2*W*⋯O30	0.88 (2)	1.87 (2)	2.745 (3)	171 (4)

Symmetry codes: (ii) 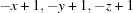; (iii) 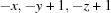.

**Table 2:** Hydrogen-bond geometry (Å, °) for **2** [Chem scheme1]

*D*—H⋯*A*	*D*—H	H⋯*A*	*D*⋯*A*	*D*—H⋯*A*
O30—H30⋯O1	0.84	1.80	2.636 (4)	178
C1—H1*B*⋯O1*W* ^ii^	0.99	2.58	3.448 (4)	146
O1*W*—H1*W*⋯O2^iii^	0.82 (2)	1.97 (2)	2.785 (4)	167 (5)
O1*W*—H2*W*⋯O30	0.86 (2)	1.95 (3)	2.759 (5)	158 (5)

Symmetry codes: (ii) 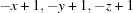; (iii) 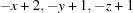.

**Table 3:** Experimental details

	**1**	**2**
Crystal data		
Chemical formula	[Gd_2_(C_28_H_28_N_4_O_2_)_2_(C_2_O_4_)]·4CH_4_O·4H_2_O	[Dy_2_(C_28_H_28_N_4_O_2_)_2_(C_2_O_4_)]·4CH_4_O·4H_2_O
*M*_r_	1507.84	1518.34
Crystal system, space group	Triclinic, *P* 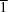	Triclinic, *P* 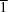
Temperature (K)	100	100
*a*, *b*, *c* (Å)	9.8778 (11), 12.8720 (16), 14.8025 (18)	9.883 (2), 12.838 (3), 14.832 (4)
α, β, γ (°)	69.092 (4), 74.786 (4), 70.324 (4)	68.213 (9), 74.653 (8), 70.552 (8)
*V* (Å^3^)	1633.7 (3)	1626.3 (7)
*Z*	1	1
Radiation type	Mo *K*α	Mo *K*α
μ (mm^−1^)	2.08	2.35
Crystal size (mm)	0.30 × 0.28 × 0.15	0.35 × 0.16 × 0.12

Data collection		
Diffractometer	Bruker D8 Venture/Photon 100 CMOS	Bruker D8 Venture/Photon 100 CMOS
Absorption correction	Multi-scan (*SADABS*; Krause *et al.*, 2015 ▸)	Multi-scan (*SADABS*; Krause *et al.*, 2015 ▸)
*T*_min_, *T*_max_	0.613, 0.746	0.629, 0.746
No. of measured, independent and observed [*I* > 2σ(*I*)] reflections	101878, 7108, 6476	93133, 7091, 6385
*R*_int_	0.052	0.059
(sin θ/λ)_max_ (Å^−1^)	0.639	0.639

Refinement		
*R*[*F* ^2^ > 2σ(*F* ^2^)], *wR*(*F* ^2^), *S*	0.020, 0.046, 1.07	0.025, 0.060, 1.06
No. of reflections	7108	7091
No. of parameters	380	380
No. of restraints	2	3
H-atom treatment	H atoms treated by a mixture of independent and constrained refinement	H atoms treated by a mixture of independent and constrained refinement
Δρ_max_, Δρ_min_ (e Å^−3^)	1.20, −0.55	1.94, −0.63

Computer programs: *APEX3* (Bruker, 2015 ▸), *SAINT* (Bruker, 2002 ▸), *SHELXT2015* (Sheldrick 2015*a* ▸), *SHELXL2017/1* (Sheldrick 2015*b* ▸), *ORTEP* (Johnson, 1976 ▸ and Farrugia, 2012 ▸), *DIAMOND* (Brandenburg, 2006 ▸), *SHELXL97* (Sheldrick, 2008 ▸) and *WinGX* (Farrugia, 1999 ▸, 2012 ▸).

## supplementary crystallographic information

### (µ-Oxalato)bis{[N,N'-bis(2-oxidobenzyl-κO)-N,N'-bis(pyridin-2-ylmethyl-κN)ethylenediamine-κ2N,N']gadolinium(III)}–methanol–water (1/4/4) (1) Crystal data

**Table:**

[Gd_2_(C_28_H_28_N_4_O_2_)_2_(C_2_O_4_)]·4CH_4_O·4H_2_O	*Z* = 1
*M**_r_* = 1507.84	*F*(000) = 764
Triclinic, *P*1	*D*_x_ = 1.533 Mg m^−^^3^
*a* = 9.8778 (11) Å	Mo *K*α radiation, λ = 0.71073 Å
*b* = 12.8720 (16) Å	Cell parameters from 9519 reflections
*c* = 14.8025 (18) Å	θ = 3.0–27.9°
α = 69.092 (4)°	µ = 2.08 mm^−^^1^
β = 74.786 (4)°	*T* = 100 K
γ = 70.324 (4)°	Prism, colourless
*V* = 1633.7 (3) Å^3^	0.30 × 0.28 × 0.14 mm

### (µ-Oxalato)bis{[N,N'-bis(2-oxidobenzyl-κO)-N,N'-bis(pyridin-2-ylmethyl-κN)ethylenediamine-κ2N,N']gadolinium(III)}–methanol–water (1/4/4) (1) Data collection

**Table:**

Bruker D8 Venture/Photon 100 CMOS diffractometer	7108 independent reflections
Radiation source: fine-focus sealed tube	6476 reflections with *I* > 2σ(*I*)
Graphite monochromator	*R*_int_ = 0.052
Detector resolution: 10.4167 pixels mm^-1^	θ_max_ = 27.0°, θ_min_ = 3.0°
φ and ω scans	*h* = −12→12
Absorption correction: multi-scan (*SADABS*; Krause *et al.*, 2015)	*k* = −16→16
*T*_min_ = 0.613, *T*_max_ = 0.746	*l* = −18→18
101878 measured reflections	

### (µ-Oxalato)bis{[N,N'-bis(2-oxidobenzyl-κO)-N,N'-bis(pyridin-2-ylmethyl-κN)ethylenediamine-κ2N,N']gadolinium(III)}–methanol–water (1/4/4) (1) Refinement

**Table:**

Refinement on *F*^2^	Primary atom site location: dual
Least-squares matrix: full	Secondary atom site location: difference Fourier map
*R*[*F*^2^ > 2σ(*F*^2^)] = 0.020	Hydrogen site location: mixed
*wR*(*F*^2^) = 0.046	H atoms treated by a mixture of independent and constrained refinement
*S* = 1.07	*w* = 1/[σ^2^(*F*_o_^2^) + (0.0132*P*)^2^ + 1.5506*P*] where *P* = (*F*_o_^2^ + 2*F*_c_^2^)/3
7108 reflections	(Δ/σ)_max_ = 0.001
380 parameters	Δρ_max_ = 1.20 e Å^−^^3^
2 restraints	Δρ_min_ = −0.55 e Å^−^^3^

### (µ-Oxalato)bis{[N,N'-bis(2-oxidobenzyl-κO)-N,N'-bis(pyridin-2-ylmethyl-κN)ethylenediamine-κ2N,N']gadolinium(III)}–methanol–water (1/4/4) (1) Special details

**Table:**

Geometry. All esds (except the esd in the dihedral angle between two l.s. planes) are estimated using the full covariance matrix. The cell esds are taken into account individually in the estimation of esds in distances, angles and torsion angles; correlations between esds in cell parameters are only used when they are defined by crystal symmetry. An approximate (isotropic) treatment of cell esds is used for estimating esds involving l.s. planes.

### (µ-Oxalato)bis{[N,N'-bis(2-oxidobenzyl-κO)-N,N'-bis(pyridin-2-ylmethyl-κN)ethylenediamine-κ2N,N']gadolinium(III)}–methanol–water (1/4/4) (1) Fractional atomic coordinates and isotropic or equivalent isotropic displacement parameters (Å^2^)

**Table:**

	*x*	*y*	*z*	*U*_iso_*/*U*_eq_	
Gd1	0.14723 (2)	0.64026 (2)	0.30503 (2)	0.02451 (4)	
N1	0.3709 (2)	0.68776 (16)	0.33127 (13)	0.0308 (4)	
N2	0.26964 (19)	0.76071 (16)	0.13842 (13)	0.0302 (4)	
N3	0.0847 (2)	0.83950 (16)	0.33400 (14)	0.0345 (4)	
N4	0.1895 (2)	0.56231 (17)	0.16178 (14)	0.0341 (4)	
O1	0.32272 (17)	0.47047 (14)	0.33368 (13)	0.0384 (4)	
O2	−0.04416 (16)	0.74756 (14)	0.22275 (11)	0.0348 (4)	
O3	0.10169 (17)	0.59622 (14)	0.47919 (11)	0.0326 (3)	
O4	0.00913 (17)	0.48457 (14)	0.62012 (11)	0.0317 (3)	
O30	0.2560 (3)	0.2853 (2)	0.46553 (17)	0.0708 (6)	
H30	0.279892	0.342451	0.422530	0.106*	
C1	0.4553 (3)	0.7393 (2)	0.23546 (17)	0.0366 (5)	
H1A	0.526548	0.676065	0.211006	0.044*	
H1B	0.510810	0.783956	0.246233	0.044*	
C2	0.3634 (3)	0.8170 (2)	0.15829 (17)	0.0356 (5)	
H2A	0.300546	0.885400	0.179071	0.043*	
H2B	0.428067	0.844484	0.096725	0.043*	
C3	0.4722 (3)	0.5828 (2)	0.38753 (18)	0.0371 (5)	
H3A	0.545817	0.607522	0.403134	0.045*	
H3B	0.415669	0.547543	0.450400	0.045*	
C4	0.5499 (3)	0.4925 (2)	0.33652 (18)	0.0376 (5)	
C5	0.7021 (3)	0.4568 (3)	0.3171 (2)	0.0530 (7)	
H5	0.757587	0.492628	0.334115	0.064*	
C6	0.7724 (3)	0.3699 (3)	0.2734 (3)	0.0662 (9)	
H6	0.875802	0.345578	0.260954	0.079*	
C7	0.6921 (3)	0.3189 (3)	0.2482 (2)	0.0595 (8)	
H7	0.740815	0.259677	0.217648	0.071*	
C8	0.5402 (3)	0.3526 (2)	0.2666 (2)	0.0473 (6)	
H8	0.485806	0.316915	0.248361	0.057*	
C9	0.4686 (3)	0.4392 (2)	0.31219 (17)	0.0359 (5)	
C10	0.1601 (3)	0.8573 (2)	0.08153 (18)	0.0361 (5)	
H10A	0.213640	0.898996	0.020397	0.043*	
H10B	0.109274	0.912135	0.120374	0.043*	
C11	0.0468 (2)	0.82508 (19)	0.05439 (17)	0.0327 (5)	
C12	0.0333 (3)	0.8522 (2)	−0.04292 (19)	0.0419 (6)	
H12	0.103411	0.884025	−0.093012	0.050*	
C13	−0.0798 (3)	0.8339 (2)	−0.0688 (2)	0.0483 (7)	
H13	−0.087322	0.852986	−0.135819	0.058*	
C14	−0.1812 (3)	0.7878 (3)	0.0040 (2)	0.0514 (7)	
H14	−0.260080	0.776115	−0.012986	0.062*	
C15	−0.1695 (3)	0.7584 (2)	0.1014 (2)	0.0438 (6)	
H15	−0.239531	0.725264	0.150492	0.053*	
C16	−0.0560 (2)	0.77657 (19)	0.12913 (17)	0.0318 (5)	
C17	0.3155 (3)	0.7667 (2)	0.39309 (19)	0.0399 (6)	
H17A	0.393828	0.801043	0.388604	0.048*	
H17B	0.293976	0.720619	0.462140	0.048*	
C18	0.1824 (3)	0.8622 (2)	0.36726 (18)	0.0379 (5)	
C19	0.1574 (4)	0.9676 (3)	0.3832 (2)	0.0555 (7)	
H19	0.230010	0.983182	0.403804	0.067*	
C20	0.0264 (4)	1.0490 (3)	0.3690 (3)	0.0643 (9)	
H20	0.006686	1.120691	0.381254	0.077*	
C21	−0.0753 (3)	1.0259 (2)	0.3370 (2)	0.0537 (7)	
H21	−0.167055	1.080485	0.327493	0.064*	
C22	−0.0409 (3)	0.9211 (2)	0.31884 (19)	0.0427 (6)	
H22	−0.110029	0.906124	0.294343	0.051*	
C23	0.3579 (3)	0.6806 (2)	0.08111 (18)	0.0379 (5)	
H23A	0.448335	0.634954	0.109017	0.046*	
H23B	0.386281	0.726014	0.012906	0.046*	
C24	0.2797 (2)	0.5989 (2)	0.07971 (17)	0.0339 (5)	
C25	0.3037 (3)	0.5614 (2)	−0.00088 (19)	0.0468 (6)	
H25	0.369457	0.587595	−0.057776	0.056*	
C26	0.2311 (3)	0.4857 (3)	0.0022 (2)	0.0563 (8)	
H26	0.245896	0.459136	−0.052756	0.068*	
C27	0.1367 (3)	0.4485 (3)	0.0856 (2)	0.0547 (7)	
H27	0.084898	0.396546	0.089189	0.066*	
C28	0.1195 (3)	0.4888 (2)	0.1637 (2)	0.0452 (6)	
H28	0.054965	0.463138	0.221597	0.054*	
C29	0.0323 (2)	0.52339 (19)	0.52893 (15)	0.0270 (4)	
C30	0.1948 (4)	0.2316 (3)	0.4227 (3)	0.0712 (10)	
H30A	0.185641	0.156599	0.468653	0.107*	
H30B	0.258399	0.220344	0.361943	0.107*	
H30C	0.098312	0.281001	0.408213	0.107*	
O1W	0.3109 (2)	0.2151 (2)	0.65369 (19)	0.0644 (6)	
H1W	0.236 (3)	0.229 (3)	0.694 (2)	0.058 (10)*	
H2W	0.290 (4)	0.245 (3)	0.5939 (16)	0.080 (13)*	

### (µ-Oxalato)bis{[N,N'-bis(2-oxidobenzyl-κO)-N,N'-bis(pyridin-2-ylmethyl-κN)ethylenediamine-κ2N,N']gadolinium(III)}–methanol–water (1/4/4) (1) Atomic displacement parameters (Å^2^)

**Table:**

	*U*^11^	*U*^22^	*U*^33^	*U*^12^	*U*^13^	*U*^23^
Gd1	0.02416 (5)	0.02753 (6)	0.02339 (6)	−0.01192 (4)	−0.00064 (4)	−0.00698 (4)
N1	0.0324 (10)	0.0350 (10)	0.0268 (9)	−0.0153 (8)	−0.0065 (7)	−0.0041 (8)
N2	0.0287 (9)	0.0350 (10)	0.0276 (10)	−0.0149 (8)	−0.0022 (7)	−0.0059 (8)
N3	0.0390 (11)	0.0297 (10)	0.0347 (11)	−0.0105 (8)	−0.0042 (8)	−0.0096 (8)
N4	0.0412 (11)	0.0324 (10)	0.0293 (10)	−0.0113 (8)	−0.0030 (8)	−0.0104 (8)
O1	0.0309 (8)	0.0337 (9)	0.0529 (11)	−0.0067 (7)	−0.0099 (7)	−0.0151 (8)
O2	0.0288 (8)	0.0438 (9)	0.0317 (9)	−0.0098 (7)	−0.0044 (6)	−0.0112 (7)
O3	0.0387 (9)	0.0427 (9)	0.0272 (8)	−0.0279 (7)	0.0007 (6)	−0.0112 (7)
O4	0.0386 (8)	0.0444 (9)	0.0215 (8)	−0.0259 (7)	−0.0012 (6)	−0.0097 (7)
O30	0.1006 (19)	0.0616 (15)	0.0601 (14)	−0.0466 (14)	−0.0135 (13)	−0.0058 (11)
C1	0.0330 (12)	0.0437 (14)	0.0358 (13)	−0.0222 (10)	−0.0053 (10)	−0.0042 (11)
C2	0.0369 (12)	0.0364 (13)	0.0316 (12)	−0.0202 (10)	−0.0003 (9)	−0.0022 (10)
C3	0.0353 (12)	0.0414 (14)	0.0365 (13)	−0.0137 (10)	−0.0160 (10)	−0.0032 (11)
C4	0.0310 (12)	0.0391 (13)	0.0351 (13)	−0.0071 (10)	−0.0087 (10)	−0.0019 (10)
C5	0.0336 (14)	0.0506 (17)	0.0624 (19)	−0.0070 (12)	−0.0130 (12)	−0.0027 (14)
C6	0.0333 (15)	0.062 (2)	0.073 (2)	0.0010 (14)	0.0003 (14)	−0.0043 (17)
C7	0.0556 (18)	0.0416 (16)	0.0500 (17)	0.0096 (14)	0.0026 (14)	−0.0065 (13)
C8	0.0543 (16)	0.0352 (14)	0.0424 (15)	−0.0020 (12)	−0.0084 (12)	−0.0091 (12)
C9	0.0345 (12)	0.0324 (12)	0.0318 (12)	−0.0021 (10)	−0.0082 (9)	−0.0033 (10)
C10	0.0411 (13)	0.0301 (12)	0.0344 (13)	−0.0143 (10)	−0.0081 (10)	−0.0006 (10)
C11	0.0339 (12)	0.0268 (11)	0.0347 (12)	−0.0047 (9)	−0.0081 (9)	−0.0073 (9)
C12	0.0500 (15)	0.0333 (13)	0.0361 (13)	−0.0061 (11)	−0.0109 (11)	−0.0047 (11)
C13	0.0592 (17)	0.0458 (15)	0.0416 (15)	−0.0034 (13)	−0.0221 (13)	−0.0149 (12)
C14	0.0476 (16)	0.0574 (18)	0.0617 (19)	−0.0079 (13)	−0.0215 (14)	−0.0290 (15)
C15	0.0333 (12)	0.0560 (16)	0.0490 (15)	−0.0134 (11)	−0.0070 (11)	−0.0220 (13)
C16	0.0293 (11)	0.0301 (11)	0.0355 (12)	−0.0030 (9)	−0.0067 (9)	−0.0127 (10)
C17	0.0421 (13)	0.0437 (14)	0.0435 (14)	−0.0163 (11)	−0.0125 (11)	−0.0156 (12)
C18	0.0475 (14)	0.0371 (13)	0.0337 (13)	−0.0179 (11)	−0.0023 (10)	−0.0127 (10)
C19	0.074 (2)	0.0444 (16)	0.0619 (19)	−0.0200 (15)	−0.0167 (16)	−0.0240 (14)
C20	0.090 (2)	0.0409 (16)	0.070 (2)	−0.0141 (16)	−0.0138 (18)	−0.0284 (16)
C21	0.0620 (18)	0.0353 (14)	0.0529 (17)	0.0005 (13)	−0.0088 (14)	−0.0135 (13)
C22	0.0426 (14)	0.0375 (14)	0.0446 (15)	−0.0077 (11)	−0.0046 (11)	−0.0128 (11)
C23	0.0324 (12)	0.0463 (14)	0.0311 (12)	−0.0132 (10)	0.0023 (9)	−0.0097 (11)
C24	0.0326 (12)	0.0341 (12)	0.0291 (12)	0.0005 (9)	−0.0064 (9)	−0.0102 (10)
C25	0.0471 (15)	0.0544 (17)	0.0348 (14)	−0.0071 (13)	0.0002 (11)	−0.0192 (12)
C26	0.0669 (19)	0.0640 (19)	0.0470 (17)	−0.0125 (15)	−0.0055 (14)	−0.0342 (15)
C27	0.0655 (19)	0.0513 (17)	0.0620 (19)	−0.0211 (14)	−0.0082 (15)	−0.0301 (15)
C28	0.0586 (16)	0.0432 (15)	0.0410 (14)	−0.0220 (13)	−0.0014 (12)	−0.0178 (12)
C29	0.0263 (10)	0.0321 (11)	0.0270 (11)	−0.0126 (9)	−0.0008 (8)	−0.0119 (9)
C30	0.094 (3)	0.0534 (19)	0.076 (2)	−0.0322 (18)	0.001 (2)	−0.0293 (18)
O1W	0.0331 (11)	0.1015 (19)	0.0682 (16)	−0.0197 (11)	−0.0019 (11)	−0.0393 (15)

### (µ-Oxalato)bis{[N,N'-bis(2-oxidobenzyl-κO)-N,N'-bis(pyridin-2-ylmethyl-κN)ethylenediamine-κ2N,N']gadolinium(III)}–methanol–water (1/4/4) (1) Geometric parameters (Å, º)

**Table:**

Gd1—O1	2.2659 (16)	C10—C11	1.501 (3)
Gd1—O2	2.2835 (16)	C10—H10A	0.9900
Gd1—O3	2.3855 (15)	C10—H10B	0.9900
Gd1—O4^i^	2.3869 (14)	C11—C12	1.388 (3)
Gd1—N4	2.5394 (19)	C11—C16	1.410 (3)
Gd1—N3	2.5926 (19)	C12—C13	1.384 (4)
Gd1—N2	2.6299 (18)	C12—H12	0.9500
Gd1—N1	2.6334 (18)	C13—C14	1.376 (4)
N1—C17	1.482 (3)	C13—H13	0.9500
N1—C1	1.492 (3)	C14—C15	1.379 (4)
N1—C3	1.498 (3)	C14—H14	0.9500
N2—C23	1.475 (3)	C15—C16	1.403 (3)
N2—C2	1.489 (3)	C15—H15	0.9500
N2—C10	1.500 (3)	C17—C18	1.492 (4)
N3—C22	1.339 (3)	C17—H17A	0.9900
N3—C18	1.343 (3)	C17—H17B	0.9900
N4—C28	1.336 (3)	C18—C19	1.389 (4)
N4—C24	1.340 (3)	C19—C20	1.374 (5)
O1—C9	1.342 (3)	C19—H19	0.9500
O2—C16	1.327 (3)	C20—C21	1.369 (5)
O3—C29	1.252 (3)	C20—H20	0.9500
O4—C29	1.248 (3)	C21—C22	1.382 (4)
O30—C30	1.429 (4)	C21—H21	0.9500
O30—H30	0.8400	C22—H22	0.9500
C1—C2	1.499 (3)	C23—C24	1.508 (3)
C1—H1A	0.9900	C23—H23A	0.9900
C1—H1B	0.9900	C23—H23B	0.9900
C2—H2A	0.9900	C24—C25	1.377 (3)
C2—H2B	0.9900	C25—C26	1.375 (4)
C3—C4	1.494 (4)	C25—H25	0.9500
C3—H3A	0.9900	C26—C27	1.377 (4)
C3—H3B	0.9900	C26—H26	0.9500
C4—C9	1.393 (4)	C27—C28	1.379 (4)
C4—C5	1.399 (3)	C27—H27	0.9500
C5—C6	1.382 (5)	C28—H28	0.9500
C5—H5	0.9500	C29—C29^i^	1.554 (4)
C6—C7	1.371 (5)	C30—H30A	0.9800
C6—H6	0.9500	C30—H30B	0.9800
C7—C8	1.395 (4)	C30—H30C	0.9800
C7—H7	0.9500	O1W—H1W	0.831 (18)
C8—C9	1.398 (4)	O1W—H2W	0.879 (18)
C8—H8	0.9500		
			
O1—Gd1—O2	144.65 (6)	C7—C8—H8	120.3
O1—Gd1—O3	83.85 (6)	C9—C8—H8	120.3
O2—Gd1—O3	117.78 (6)	O1—C9—C4	119.8 (2)
O1—Gd1—O4^i^	82.26 (6)	O1—C9—C8	120.6 (2)
O2—Gd1—O4^i^	81.09 (6)	C4—C9—C8	119.6 (2)
O3—Gd1—O4^i^	68.12 (5)	N2—C10—C11	117.04 (19)
O1—Gd1—N4	72.72 (6)	N2—C10—H10A	108.0
O2—Gd1—N4	74.51 (6)	C11—C10—H10A	108.0
O3—Gd1—N4	145.21 (6)	N2—C10—H10B	108.0
O4^i^—Gd1—N4	83.25 (6)	C11—C10—H10B	108.0
O1—Gd1—N3	137.96 (6)	H10A—C10—H10B	107.3
O2—Gd1—N3	76.82 (6)	C12—C11—C16	119.5 (2)
O3—Gd1—N3	76.31 (6)	C12—C11—C10	120.9 (2)
O4^i^—Gd1—N3	121.96 (6)	C16—C11—C10	119.3 (2)
N4—Gd1—N3	137.77 (6)	C13—C12—C11	121.6 (3)
O1—Gd1—N2	101.15 (6)	C13—C12—H12	119.2
O2—Gd1—N2	76.69 (6)	C11—C12—H12	119.2
O3—Gd1—N2	146.18 (5)	C14—C13—C12	119.0 (3)
O4^i^—Gd1—N2	145.50 (5)	C14—C13—H13	120.5
N4—Gd1—N2	65.63 (6)	C12—C13—H13	120.5
N3—Gd1—N2	78.07 (6)	C13—C14—C15	120.7 (3)
O1—Gd1—N1	74.44 (6)	C13—C14—H14	119.6
O2—Gd1—N1	133.51 (6)	C15—C14—H14	119.6
O3—Gd1—N1	80.10 (5)	C14—C15—C16	121.1 (3)
O4^i^—Gd1—N1	142.30 (5)	C14—C15—H15	119.4
N4—Gd1—N1	116.30 (6)	C16—C15—H15	119.4
N3—Gd1—N1	65.93 (6)	O2—C16—C15	121.1 (2)
N2—Gd1—N1	69.43 (6)	O2—C16—C11	120.8 (2)
C17—N1—C1	111.49 (19)	C15—C16—C11	118.0 (2)
C17—N1—C3	105.29 (18)	N1—C17—C18	115.3 (2)
C1—N1—C3	108.47 (18)	N1—C17—H17A	108.5
C17—N1—Gd1	108.34 (13)	C18—C17—H17A	108.5
C1—N1—Gd1	110.98 (13)	N1—C17—H17B	108.5
C3—N1—Gd1	112.16 (13)	C18—C17—H17B	108.5
C23—N2—C2	110.38 (18)	H17A—C17—H17B	107.5
C23—N2—C10	110.90 (18)	N3—C18—C19	121.8 (3)
C2—N2—C10	105.60 (17)	N3—C18—C17	117.3 (2)
C23—N2—Gd1	107.83 (13)	C19—C18—C17	120.8 (2)
C2—N2—Gd1	109.63 (13)	C20—C19—C18	119.3 (3)
C10—N2—Gd1	112.50 (13)	C20—C19—H19	120.4
C22—N3—C18	117.8 (2)	C18—C19—H19	120.4
C22—N3—Gd1	123.69 (17)	C21—C20—C19	119.4 (3)
C18—N3—Gd1	118.52 (15)	C21—C20—H20	120.3
C28—N4—C24	118.3 (2)	C19—C20—H20	120.3
C28—N4—Gd1	121.90 (16)	C20—C21—C22	118.2 (3)
C24—N4—Gd1	119.79 (15)	C20—C21—H21	120.9
C9—O1—Gd1	135.00 (15)	C22—C21—H21	120.9
C16—O2—Gd1	131.61 (13)	N3—C22—C21	123.4 (3)
C29—O3—Gd1	119.02 (13)	N3—C22—H22	118.3
C29—O4—Gd1^i^	119.12 (13)	C21—C22—H22	118.3
C30—O30—H30	109.5	N2—C23—C24	113.32 (18)
N1—C1—C2	114.19 (18)	N2—C23—H23A	108.9
N1—C1—H1A	108.7	C24—C23—H23A	108.9
C2—C1—H1A	108.7	N2—C23—H23B	108.9
N1—C1—H1B	108.7	C24—C23—H23B	108.9
C2—C1—H1B	108.7	H23A—C23—H23B	107.7
H1A—C1—H1B	107.6	N4—C24—C25	122.1 (2)
N2—C2—C1	113.77 (19)	N4—C24—C23	116.5 (2)
N2—C2—H2A	108.8	C25—C24—C23	121.4 (2)
C1—C2—H2A	108.8	C26—C25—C24	119.0 (3)
N2—C2—H2B	108.8	C26—C25—H25	120.5
C1—C2—H2B	108.8	C24—C25—H25	120.5
H2A—C2—H2B	107.7	C25—C26—C27	119.5 (3)
C4—C3—N1	115.32 (19)	C25—C26—H26	120.2
C4—C3—H3A	108.4	C27—C26—H26	120.2
N1—C3—H3A	108.4	C26—C27—C28	118.1 (3)
C4—C3—H3B	108.4	C26—C27—H27	120.9
N1—C3—H3B	108.4	C28—C27—H27	120.9
H3A—C3—H3B	107.5	N4—C28—C27	123.0 (3)
C9—C4—C5	119.6 (3)	N4—C28—H28	118.5
C9—C4—C3	119.0 (2)	C27—C28—H28	118.5
C5—C4—C3	121.3 (2)	O4—C29—O3	126.83 (19)
C6—C5—C4	120.6 (3)	O4—C29—C29^i^	116.6 (2)
C6—C5—H5	119.7	O3—C29—C29^i^	116.6 (2)
C4—C5—H5	119.7	O30—C30—H30A	109.5
C7—C6—C5	119.6 (3)	O30—C30—H30B	109.5
C7—C6—H6	120.2	H30A—C30—H30B	109.5
C5—C6—H6	120.2	O30—C30—H30C	109.5
C6—C7—C8	121.1 (3)	H30A—C30—H30C	109.5
C6—C7—H7	119.4	H30B—C30—H30C	109.5
C8—C7—H7	119.4	H1W—O1W—H2W	110 (3)
C7—C8—C9	119.4 (3)		
			
C17—N1—C1—C2	−85.0 (2)	C10—C11—C16—O2	−7.4 (3)
C3—N1—C1—C2	159.5 (2)	C12—C11—C16—C15	−0.5 (3)
Gd1—N1—C1—C2	35.9 (2)	C10—C11—C16—C15	173.5 (2)
C23—N2—C2—C1	−74.8 (2)	C1—N1—C17—C18	79.1 (2)
C10—N2—C2—C1	165.3 (2)	C3—N1—C17—C18	−163.5 (2)
Gd1—N2—C2—C1	43.8 (2)	Gd1—N1—C17—C18	−43.3 (2)
N1—C1—C2—N2	−56.1 (3)	C22—N3—C18—C19	−1.7 (4)
C17—N1—C3—C4	−176.3 (2)	Gd1—N3—C18—C19	179.1 (2)
C1—N1—C3—C4	−56.9 (2)	C22—N3—C18—C17	174.2 (2)
Gd1—N1—C3—C4	66.1 (2)	Gd1—N3—C18—C17	−5.0 (3)
N1—C3—C4—C9	−61.9 (3)	N1—C17—C18—N3	34.3 (3)
N1—C3—C4—C5	121.1 (2)	N1—C17—C18—C19	−149.8 (2)
C9—C4—C5—C6	0.5 (4)	N3—C18—C19—C20	3.0 (4)
C3—C4—C5—C6	177.4 (3)	C17—C18—C19—C20	−172.8 (3)
C4—C5—C6—C7	0.5 (5)	C18—C19—C20—C21	−1.6 (5)
C5—C6—C7—C8	−0.6 (5)	C19—C20—C21—C22	−0.9 (5)
C6—C7—C8—C9	−0.4 (4)	C18—N3—C22—C21	−1.0 (4)
Gd1—O1—C9—C4	52.5 (3)	Gd1—N3—C22—C21	178.2 (2)
Gd1—O1—C9—C8	−128.3 (2)	C20—C21—C22—N3	2.3 (4)
C5—C4—C9—O1	177.8 (2)	C2—N2—C23—C24	165.50 (19)
C3—C4—C9—O1	0.8 (3)	C10—N2—C23—C24	−77.8 (2)
C5—C4—C9—C8	−1.5 (4)	Gd1—N2—C23—C24	45.8 (2)
C3—C4—C9—C8	−178.4 (2)	C28—N4—C24—C25	−1.2 (4)
C7—C8—C9—O1	−177.8 (2)	Gd1—N4—C24—C25	−178.42 (18)
C7—C8—C9—C4	1.4 (4)	C28—N4—C24—C23	−179.2 (2)
C23—N2—C10—C11	62.5 (3)	Gd1—N4—C24—C23	3.6 (3)
C2—N2—C10—C11	−177.9 (2)	N2—C23—C24—N4	−35.2 (3)
Gd1—N2—C10—C11	−58.3 (2)	N2—C23—C24—C25	146.8 (2)
N2—C10—C11—C12	−120.9 (2)	N4—C24—C25—C26	1.1 (4)
N2—C10—C11—C16	65.2 (3)	C23—C24—C25—C26	179.1 (3)
C16—C11—C12—C13	0.7 (4)	C24—C25—C26—C27	−0.3 (4)
C10—C11—C12—C13	−173.2 (2)	C25—C26—C27—C28	−0.4 (5)
C11—C12—C13—C14	0.0 (4)	C24—N4—C28—C27	0.5 (4)
C12—C13—C14—C15	−1.0 (4)	Gd1—N4—C28—C27	177.6 (2)
C13—C14—C15—C16	1.2 (4)	C26—C27—C28—N4	0.3 (5)
Gd1—O2—C16—C15	128.8 (2)	Gd1^i^—O4—C29—O3	174.05 (18)
Gd1—O2—C16—C11	−50.2 (3)	Gd1^i^—O4—C29—C29^i^	−5.8 (3)
C14—C15—C16—O2	−179.5 (2)	Gd1—O3—C29—O4	174.16 (17)
C14—C15—C16—C11	−0.5 (4)	Gd1—O3—C29—C29^i^	−6.0 (3)
C12—C11—C16—O2	178.6 (2)		

Symmetry code: (i) −*x*, −*y*+1, −*z*+1.

### (µ-Oxalato)bis{[N,N'-bis(2-oxidobenzyl-κO)-N,N'-bis(pyridin-2-ylmethyl-κN)ethylenediamine-κ2N,N']gadolinium(III)}–methanol–water (1/4/4) (1) Hydrogen-bond geometry (Å, º)

**Table:**

*D*—H···*A*	*D*—H	H···*A*	*D*···*A*	*D*—H···*A*
O30—H30···O1	0.84	1.80	2.643 (3)	177
C1—H1*B*···O1*W*^ii^	0.99	2.59	3.459 (3)	147
O1*W*—H1*W*···O2^i^	0.83 (2)	1.96 (2)	2.786 (3)	170 (3)
O1*W*—H2*W*···O30	0.88 (2)	1.87 (2)	2.745 (3)	171 (4)

Symmetry codes: (i) −*x*, −*y*+1, −*z*+1; (ii) −*x*+1, −*y*+1, −*z*+1.

### (µ-Oxalato)bis{[N,N'-bis(2-oxidobenzyl-κO)-N,N'-bis(pyridin-2-ylmethyl-κN)ethylenediamine-κ2N,N']dysprosium(III)}–methanol–water (1/4/4) (2) Crystal data

**Table:**

[Dy_2_(C_28_H_28_N_4_O_2_)_2_(C_2_O_4_)]·4CH_4_O·4H_2_O	*Z* = 1
*M**_r_* = 1518.34	*F*(000) = 768
Triclinic, *P*1	*D*_x_ = 1.550 Mg m^−^^3^
*a* = 9.883 (2) Å	Mo *K*α radiation, λ = 0.71073 Å
*b* = 12.838 (3) Å	Cell parameters from 9284 reflections
*c* = 14.832 (4) Å	θ = 3.0–27.7°
α = 68.213 (9)°	µ = 2.35 mm^−^^1^
β = 74.653 (8)°	*T* = 100 K
γ = 70.552 (8)°	Prism, colourless
*V* = 1626.3 (7) Å^3^	0.35 × 0.16 × 0.12 mm

### (µ-Oxalato)bis{[N,N'-bis(2-oxidobenzyl-κO)-N,N'-bis(pyridin-2-ylmethyl-κN)ethylenediamine-κ2N,N']dysprosium(III)}–methanol–water (1/4/4) (2) Data collection

**Table:**

Bruker D8 Venture/Photon 100 CMOS diffractometer	7091 independent reflections
Radiation source: fine-focus sealed tube	6385 reflections with *I* > 2σ(*I*)
Graphite monochromator	*R*_int_ = 0.059
Detector resolution: 10.4167 pixels mm^-1^	θ_max_ = 27.0°, θ_min_ = 3.0°
φ and ω scans	*h* = −12→12
Absorption correction: multi-scan (*SADABS*; Krause *et al.*, 2015)	*k* = −16→16
*T*_min_ = 0.629, *T*_max_ = 0.746	*l* = −18→18
93133 measured reflections	

### (µ-Oxalato)bis{[N,N'-bis(2-oxidobenzyl-κO)-N,N'-bis(pyridin-2-ylmethyl-κN)ethylenediamine-κ2N,N']dysprosium(III)}–methanol–water (1/4/4) (2) Refinement

**Table:**

Refinement on *F*^2^	Primary atom site location: dual
Least-squares matrix: full	Secondary atom site location: difference Fourier map
*R*[*F*^2^ > 2σ(*F*^2^)] = 0.025	Hydrogen site location: mixed
*wR*(*F*^2^) = 0.060	H atoms treated by a mixture of independent and constrained refinement
*S* = 1.06	*w* = 1/[σ^2^(*F*_o_^2^) + (0.0244*P*)^2^ + 1.9868*P*] where *P* = (*F*_o_^2^ + 2*F*_c_^2^)/3
7091 reflections	(Δ/σ)_max_ = 0.002
380 parameters	Δρ_max_ = 1.94 e Å^−^^3^
3 restraints	Δρ_min_ = −0.63 e Å^−^^3^

### (µ-Oxalato)bis{[N,N'-bis(2-oxidobenzyl-κO)-N,N'-bis(pyridin-2-ylmethyl-κN)ethylenediamine-κ2N,N']dysprosium(III)}–methanol–water (1/4/4) (2) Special details

**Table:**

Geometry. All esds (except the esd in the dihedral angle between two l.s. planes) are estimated using the full covariance matrix. The cell esds are taken into account individually in the estimation of esds in distances, angles and torsion angles; correlations between esds in cell parameters are only used when they are defined by crystal symmetry. An approximate (isotropic) treatment of cell esds is used for estimating esds involving l.s. planes.

### (µ-Oxalato)bis{[N,N'-bis(2-oxidobenzyl-κO)-N,N'-bis(pyridin-2-ylmethyl-κN)ethylenediamine-κ2N,N']dysprosium(III)}–methanol–water (1/4/4) (2) Fractional atomic coordinates and isotropic or equivalent isotropic displacement parameters (Å^2^)

**Table:**

	*x*	*y*	*z*	*U*_iso_*/*U*_eq_	
Dy1	0.85062 (2)	0.36072 (2)	0.69594 (2)	0.02699 (5)	
N1	0.6306 (3)	0.3122 (2)	0.66909 (18)	0.0352 (5)	
N2	0.7282 (3)	0.2394 (2)	0.86207 (18)	0.0351 (5)	
N3	0.9135 (3)	0.1612 (2)	0.66925 (19)	0.0383 (6)	
N4	0.8096 (3)	0.4397 (2)	0.83619 (18)	0.0370 (6)	
O1	0.6811 (2)	0.52962 (19)	0.66391 (17)	0.0430 (5)	
O2	1.0390 (2)	0.25545 (19)	0.77711 (15)	0.0370 (5)	
O3	0.8993 (2)	0.40098 (19)	0.52306 (14)	0.0370 (5)	
O4	0.9940 (2)	0.51292 (19)	0.38014 (14)	0.0362 (5)	
O30	0.7330 (4)	0.7243 (3)	0.5351 (2)	0.0764 (9)	
H30	0.718496	0.661941	0.576725	0.115*	
C1	0.5449 (3)	0.2587 (3)	0.7655 (2)	0.0439 (8)	
H1A	0.472908	0.321693	0.789304	0.053*	
H1B	0.490545	0.213183	0.754747	0.053*	
C2	0.6350 (4)	0.1817 (3)	0.8429 (2)	0.0420 (7)	
H2A	0.698079	0.113165	0.823044	0.050*	
H2B	0.570144	0.153596	0.904753	0.050*	
C3	0.5280 (3)	0.4178 (3)	0.6129 (2)	0.0425 (7)	
H3A	0.583081	0.454137	0.548988	0.051*	
H3B	0.453500	0.392579	0.598837	0.051*	
C4	0.4534 (3)	0.5066 (3)	0.6633 (2)	0.0429 (7)	
C5	0.3007 (4)	0.5435 (4)	0.6832 (3)	0.0612 (10)	
H5	0.244013	0.508835	0.666133	0.073*	
C6	0.2325 (5)	0.6292 (4)	0.7271 (4)	0.0755 (14)	
H6	0.129331	0.653494	0.739876	0.091*	
C7	0.3137 (5)	0.6797 (4)	0.7526 (3)	0.0699 (13)	
H7	0.266094	0.738051	0.783716	0.084*	
C8	0.4657 (4)	0.6458 (3)	0.7330 (3)	0.0541 (9)	
H8	0.521300	0.680638	0.750940	0.065*	
C9	0.5350 (3)	0.5603 (3)	0.6870 (2)	0.0414 (7)	
C10	0.8356 (4)	0.1433 (3)	0.9201 (2)	0.0403 (7)	
H10A	0.885352	0.087425	0.882625	0.048*	
H10B	0.781416	0.102000	0.982074	0.048*	
C11	0.9506 (3)	0.1755 (3)	0.9460 (2)	0.0367 (6)	
C12	0.9649 (4)	0.1478 (3)	1.0434 (2)	0.0462 (8)	
H12	0.894743	0.115714	1.094305	0.055*	
C13	1.0791 (4)	0.1657 (3)	1.0681 (3)	0.0538 (9)	
H13	1.087727	0.145829	1.135118	0.065*	
C14	1.1796 (4)	0.2126 (3)	0.9944 (3)	0.0552 (9)	
H14	1.259234	0.224058	1.010739	0.066*	
C15	1.1668 (4)	0.2435 (3)	0.8968 (3)	0.0458 (8)	
H15	1.236347	0.277543	0.846914	0.055*	
C16	1.0523 (3)	0.2253 (3)	0.8705 (2)	0.0354 (6)	
C17	0.6850 (4)	0.2350 (3)	0.6072 (2)	0.0435 (7)	
H17A	0.706153	0.282658	0.537894	0.052*	
H17B	0.606877	0.200092	0.611962	0.052*	
C18	0.8178 (4)	0.1396 (3)	0.6332 (2)	0.0423 (7)	
C19	0.8446 (5)	0.0344 (3)	0.6155 (3)	0.0616 (10)	
H19	0.773484	0.019132	0.593305	0.074*	
C20	0.9743 (5)	−0.0461 (4)	0.6304 (3)	0.0690 (12)	
H20	0.995152	−0.117133	0.616886	0.083*	
C21	1.0750 (5)	−0.0245 (3)	0.6651 (3)	0.0605 (10)	
H21	1.166397	−0.079264	0.675028	0.073*	
C22	1.0391 (4)	0.0793 (3)	0.6851 (3)	0.0477 (8)	
H22	1.106771	0.093468	0.711293	0.057*	
C23	0.6396 (3)	0.3207 (3)	0.9182 (2)	0.0432 (7)	
H23A	0.609733	0.275136	0.986865	0.052*	
H23B	0.550307	0.367032	0.889121	0.052*	
C24	0.7191 (3)	0.4020 (3)	0.9188 (2)	0.0382 (7)	
C25	0.6957 (4)	0.4392 (3)	0.9992 (3)	0.0525 (9)	
H25	0.629682	0.412743	1.056475	0.063*	
C26	0.7693 (5)	0.5151 (4)	0.9953 (3)	0.0623 (10)	
H26	0.756092	0.540626	1.050250	0.075*	
C27	0.8620 (5)	0.5537 (4)	0.9110 (3)	0.0611 (10)	
H27	0.912648	0.607246	0.906183	0.073*	
C28	0.8803 (4)	0.5130 (3)	0.8334 (3)	0.0491 (8)	
H28	0.945997	0.538471	0.775576	0.059*	
C29	0.9690 (3)	0.4748 (3)	0.4720 (2)	0.0312 (6)	
C30	0.8049 (6)	0.7736 (4)	0.5747 (4)	0.0874 (16)	
H30A	0.794694	0.855829	0.536520	0.131*	
H30B	0.908275	0.732038	0.571185	0.131*	
H30C	0.761049	0.766725	0.643423	0.131*	
O1W	0.6925 (3)	0.7827 (4)	0.3435 (3)	0.0742 (9)	
H1W	0.768 (3)	0.763 (4)	0.307 (3)	0.090 (17)*	
H2W	0.711 (6)	0.746 (4)	0.4022 (18)	0.10 (2)*	

### (µ-Oxalato)bis{[N,N'-bis(2-oxidobenzyl-κO)-N,N'-bis(pyridin-2-ylmethyl-κN)ethylenediamine-κ2N,N']dysprosium(III)}–methanol–water (1/4/4) (2) Atomic displacement parameters (Å^2^)

**Table:**

	*U*^11^	*U*^22^	*U*^33^	*U*^12^	*U*^13^	*U*^23^
Dy1	0.02658 (7)	0.03170 (7)	0.02368 (7)	−0.01256 (5)	0.00064 (4)	−0.00858 (5)
N1	0.0363 (13)	0.0385 (14)	0.0313 (12)	−0.0163 (11)	−0.0087 (10)	−0.0036 (10)
N2	0.0336 (12)	0.0423 (14)	0.0304 (12)	−0.0180 (11)	−0.0002 (10)	−0.0087 (11)
N3	0.0425 (14)	0.0350 (13)	0.0359 (14)	−0.0110 (11)	−0.0056 (11)	−0.0093 (11)
N4	0.0407 (14)	0.0359 (13)	0.0323 (13)	−0.0075 (11)	−0.0023 (10)	−0.0128 (11)
O1	0.0384 (12)	0.0367 (12)	0.0539 (14)	−0.0058 (9)	−0.0119 (10)	−0.0147 (10)
O2	0.0304 (10)	0.0478 (12)	0.0321 (11)	−0.0086 (9)	−0.0052 (8)	−0.0131 (9)
O3	0.0450 (12)	0.0477 (12)	0.0279 (10)	−0.0287 (10)	0.0019 (9)	−0.0130 (9)
O4	0.0443 (11)	0.0502 (12)	0.0235 (10)	−0.0289 (10)	0.0004 (8)	−0.0114 (9)
O30	0.101 (2)	0.0593 (18)	0.070 (2)	−0.0406 (18)	−0.0105 (18)	−0.0069 (15)
C1	0.0382 (16)	0.055 (2)	0.0406 (17)	−0.0265 (15)	−0.0046 (13)	−0.0054 (15)
C2	0.0447 (17)	0.0437 (18)	0.0367 (16)	−0.0230 (15)	−0.0010 (13)	−0.0054 (14)
C3	0.0405 (17)	0.0515 (19)	0.0359 (16)	−0.0180 (15)	−0.0163 (13)	−0.0018 (14)
C4	0.0380 (16)	0.0435 (18)	0.0363 (17)	−0.0063 (14)	−0.0113 (13)	−0.0005 (14)
C5	0.0369 (18)	0.064 (3)	0.065 (3)	−0.0081 (18)	−0.0109 (17)	−0.004 (2)
C6	0.041 (2)	0.069 (3)	0.079 (3)	0.005 (2)	0.000 (2)	−0.005 (2)
C7	0.065 (3)	0.051 (2)	0.054 (2)	0.014 (2)	0.002 (2)	−0.0050 (19)
C8	0.059 (2)	0.0417 (19)	0.047 (2)	0.0006 (17)	−0.0085 (17)	−0.0100 (16)
C9	0.0391 (16)	0.0387 (17)	0.0331 (16)	−0.0033 (13)	−0.0080 (13)	−0.0013 (13)
C10	0.0472 (18)	0.0344 (16)	0.0356 (16)	−0.0159 (14)	−0.0071 (13)	−0.0021 (13)
C11	0.0384 (16)	0.0292 (15)	0.0391 (16)	−0.0056 (12)	−0.0068 (13)	−0.0093 (12)
C12	0.055 (2)	0.0376 (17)	0.0387 (17)	−0.0062 (15)	−0.0128 (15)	−0.0054 (14)
C13	0.067 (2)	0.052 (2)	0.046 (2)	−0.0057 (18)	−0.0245 (18)	−0.0162 (17)
C14	0.052 (2)	0.063 (2)	0.061 (2)	−0.0081 (18)	−0.0208 (18)	−0.029 (2)
C15	0.0351 (16)	0.059 (2)	0.0495 (19)	−0.0113 (15)	−0.0069 (14)	−0.0251 (17)
C16	0.0334 (15)	0.0344 (15)	0.0368 (16)	−0.0017 (12)	−0.0071 (12)	−0.0147 (13)
C17	0.0513 (19)	0.0443 (18)	0.0423 (18)	−0.0168 (15)	−0.0141 (15)	−0.0132 (15)
C18	0.0545 (19)	0.0415 (17)	0.0352 (16)	−0.0192 (15)	−0.0043 (14)	−0.0129 (14)
C19	0.083 (3)	0.051 (2)	0.064 (2)	−0.019 (2)	−0.016 (2)	−0.0283 (19)
C20	0.096 (3)	0.048 (2)	0.069 (3)	−0.014 (2)	−0.011 (2)	−0.032 (2)
C21	0.069 (3)	0.042 (2)	0.056 (2)	0.0030 (18)	−0.0085 (19)	−0.0170 (18)
C22	0.0507 (19)	0.0397 (18)	0.048 (2)	−0.0061 (15)	−0.0073 (15)	−0.0143 (15)
C23	0.0352 (16)	0.054 (2)	0.0325 (16)	−0.0134 (14)	0.0043 (12)	−0.0098 (14)
C24	0.0368 (15)	0.0381 (16)	0.0290 (15)	0.0035 (13)	−0.0053 (12)	−0.0105 (13)
C25	0.054 (2)	0.061 (2)	0.0399 (19)	−0.0074 (18)	−0.0015 (15)	−0.0234 (17)
C26	0.075 (3)	0.071 (3)	0.048 (2)	−0.012 (2)	−0.0042 (19)	−0.036 (2)
C27	0.073 (3)	0.060 (2)	0.064 (3)	−0.023 (2)	−0.004 (2)	−0.035 (2)
C28	0.061 (2)	0.0468 (19)	0.0449 (19)	−0.0197 (17)	−0.0011 (16)	−0.0204 (16)
C29	0.0299 (14)	0.0376 (15)	0.0295 (14)	−0.0142 (12)	0.0009 (11)	−0.0134 (12)
C30	0.111 (4)	0.073 (3)	0.093 (4)	−0.046 (3)	0.016 (3)	−0.044 (3)
O1W	0.0373 (14)	0.123 (3)	0.079 (2)	−0.0221 (16)	0.0020 (15)	−0.056 (2)

### (µ-Oxalato)bis{[N,N'-bis(2-oxidobenzyl-κO)-N,N'-bis(pyridin-2-ylmethyl-κN)ethylenediamine-κ2N,N']dysprosium(III)}–methanol–water (1/4/4) (2) Geometric parameters (Å, º)

**Table:**

Dy1—O1	2.230 (2)	C10—C11	1.508 (4)
Dy1—O2	2.246 (2)	C10—H10A	0.9900
Dy1—O3	2.367 (2)	C10—H10B	0.9900
Dy1—O4^i^	2.3762 (19)	C11—C12	1.388 (5)
Dy1—N4	2.523 (3)	C11—C16	1.405 (4)
Dy1—N3	2.581 (3)	C12—C13	1.384 (5)
Dy1—N2	2.606 (2)	C12—H12	0.9500
Dy1—N1	2.612 (2)	C13—C14	1.371 (6)
N1—C17	1.473 (4)	C13—H13	0.9500
N1—C1	1.500 (4)	C14—C15	1.381 (5)
N1—C3	1.501 (4)	C14—H14	0.9500
N2—C23	1.483 (4)	C15—C16	1.402 (4)
N2—C10	1.488 (4)	C15—H15	0.9500
N2—C2	1.492 (4)	C17—C18	1.490 (5)
N3—C22	1.343 (4)	C17—H17A	0.9900
N3—C18	1.345 (4)	C17—H17B	0.9900
N4—C28	1.330 (4)	C18—C19	1.398 (5)
N4—C24	1.343 (4)	C19—C20	1.363 (6)
O1—C9	1.348 (4)	C19—H19	0.9500
O2—C16	1.324 (4)	C20—C21	1.376 (6)
O3—C29	1.254 (3)	C20—H20	0.9500
O4—C29	1.251 (3)	C21—C22	1.384 (5)
O30—C30	1.431 (6)	C21—H21	0.9500
O30—H30	0.8400	C22—H22	0.9500
C1—C2	1.483 (5)	C23—C24	1.506 (5)
C1—H1A	0.9900	C23—H23A	0.9900
C1—H1B	0.9900	C23—H23B	0.9900
C2—H2A	0.9900	C24—C25	1.381 (5)
C2—H2B	0.9900	C25—C26	1.374 (6)
C3—C4	1.479 (5)	C25—H25	0.9500
C3—H3A	0.9900	C26—C27	1.373 (6)
C3—H3B	0.9900	C26—H26	0.9500
C4—C9	1.396 (5)	C27—C28	1.379 (5)
C4—C5	1.407 (5)	C27—H27	0.9500
C5—C6	1.376 (7)	C28—H28	0.9500
C5—H5	0.9500	C29—C29^i^	1.555 (5)
C6—C7	1.377 (7)	C30—H30A	0.9800
C6—H6	0.9500	C30—H30B	0.9800
C7—C8	1.400 (6)	C30—H30C	0.9800
C7—H7	0.9500	O1W—H1W	0.824 (19)
C8—C9	1.395 (5)	O1W—H2W	0.859 (19)
C8—H8	0.9500		
			
O1—Dy1—O2	144.63 (8)	C9—C8—H8	120.3
O1—Dy1—O3	84.42 (8)	C7—C8—H8	120.3
O2—Dy1—O3	116.58 (8)	O1—C9—C8	120.5 (3)
O1—Dy1—O4^i^	81.35 (8)	O1—C9—C4	119.3 (3)
O2—Dy1—O4^i^	80.87 (8)	C8—C9—C4	120.2 (3)
O3—Dy1—O4^i^	68.63 (6)	N2—C10—C11	117.3 (2)
O1—Dy1—N4	72.89 (8)	N2—C10—H10A	108.0
O2—Dy1—N4	74.69 (8)	C11—C10—H10A	108.0
O3—Dy1—N4	145.79 (8)	N2—C10—H10B	108.0
O4^i^—Dy1—N4	82.64 (8)	C11—C10—H10B	108.0
O1—Dy1—N3	138.31 (8)	H10A—C10—H10B	107.2
O2—Dy1—N3	76.73 (8)	C12—C11—C16	119.5 (3)
O3—Dy1—N3	75.05 (8)	C12—C11—C10	120.9 (3)
O4^i^—Dy1—N3	121.88 (8)	C16—C11—C10	119.4 (3)
N4—Dy1—N3	138.23 (8)	C13—C12—C11	121.5 (3)
O1—Dy1—N2	102.00 (8)	C13—C12—H12	119.2
O2—Dy1—N2	77.22 (8)	C11—C12—H12	119.2
O3—Dy1—N2	145.35 (7)	C14—C13—C12	119.0 (3)
O4^i^—Dy1—N2	145.73 (7)	C14—C13—H13	120.5
N4—Dy1—N2	66.38 (8)	C12—C13—H13	120.5
N3—Dy1—N2	78.08 (8)	C13—C14—C15	120.9 (3)
O1—Dy1—N1	75.09 (8)	C13—C14—H14	119.6
O2—Dy1—N1	133.82 (8)	C15—C14—H14	119.6
O3—Dy1—N1	79.56 (7)	C14—C15—C16	120.8 (3)
O4^i^—Dy1—N1	141.93 (7)	C14—C15—H15	119.6
N4—Dy1—N1	117.22 (8)	C16—C15—H15	119.6
N3—Dy1—N1	65.83 (8)	O2—C16—C15	121.0 (3)
N2—Dy1—N1	69.63 (8)	O2—C16—C11	120.7 (3)
C17—N1—C1	111.6 (3)	C15—C16—C11	118.3 (3)
C17—N1—C3	105.0 (2)	N1—C17—C18	114.9 (3)
C1—N1—C3	107.6 (2)	N1—C17—H17A	108.5
C17—N1—Dy1	108.94 (18)	C18—C17—H17A	108.5
C1—N1—Dy1	111.06 (17)	N1—C17—H17B	108.5
C3—N1—Dy1	112.49 (17)	C18—C17—H17B	108.5
C23—N2—C10	110.9 (2)	H17A—C17—H17B	107.5
C23—N2—C2	110.7 (2)	N3—C18—C19	121.8 (3)
C10—N2—C2	105.4 (2)	N3—C18—C17	117.4 (3)
C23—N2—Dy1	107.39 (18)	C19—C18—C17	120.7 (3)
C10—N2—Dy1	112.67 (17)	C20—C19—C18	119.1 (4)
C2—N2—Dy1	109.85 (17)	C20—C19—H19	120.5
C22—N3—C18	117.8 (3)	C18—C19—H19	120.5
C22—N3—Dy1	123.9 (2)	C19—C20—C21	120.0 (4)
C18—N3—Dy1	118.3 (2)	C19—C20—H20	120.0
C28—N4—C24	118.4 (3)	C21—C20—H20	120.0
C28—N4—Dy1	122.3 (2)	C20—C21—C22	118.0 (4)
C24—N4—Dy1	119.1 (2)	C20—C21—H21	121.0
C9—O1—Dy1	133.9 (2)	C22—C21—H21	121.0
C16—O2—Dy1	132.11 (18)	N3—C22—C21	123.4 (4)
C29—O3—Dy1	118.66 (17)	N3—C22—H22	118.3
C29—O4—Dy1^i^	119.02 (17)	C21—C22—H22	118.3
C30—O30—H30	109.5	N2—C23—C24	113.1 (2)
C2—C1—N1	114.0 (3)	N2—C23—H23A	109.0
C2—C1—H1A	108.8	C24—C23—H23A	109.0
N1—C1—H1A	108.8	N2—C23—H23B	109.0
C2—C1—H1B	108.8	C24—C23—H23B	109.0
N1—C1—H1B	108.8	H23A—C23—H23B	107.8
H1A—C1—H1B	107.7	N4—C24—C25	121.9 (3)
C1—C2—N2	113.7 (3)	N4—C24—C23	116.7 (3)
C1—C2—H2A	108.8	C25—C24—C23	121.4 (3)
N2—C2—H2A	108.8	C26—C25—C24	119.1 (3)
C1—C2—H2B	108.8	C26—C25—H25	120.5
N2—C2—H2B	108.8	C24—C25—H25	120.5
H2A—C2—H2B	107.7	C27—C26—C25	119.2 (3)
C4—C3—N1	115.0 (3)	C27—C26—H26	120.4
C4—C3—H3A	108.5	C25—C26—H26	120.4
N1—C3—H3A	108.5	C26—C27—C28	118.7 (4)
C4—C3—H3B	108.5	C26—C27—H27	120.7
N1—C3—H3B	108.5	C28—C27—H27	120.7
H3A—C3—H3B	107.5	N4—C28—C27	122.7 (3)
C9—C4—C5	118.8 (3)	N4—C28—H28	118.7
C9—C4—C3	119.7 (3)	C27—C28—H28	118.7
C5—C4—C3	121.4 (3)	O4—C29—O3	126.9 (3)
C6—C5—C4	120.9 (4)	O4—C29—C29^i^	116.1 (3)
C6—C5—H5	119.5	O3—C29—C29^i^	117.0 (3)
C4—C5—H5	119.5	O30—C30—H30A	109.5
C5—C6—C7	120.0 (4)	O30—C30—H30B	109.5
C5—C6—H6	120.0	H30A—C30—H30B	109.5
C7—C6—H6	120.0	O30—C30—H30C	109.5
C6—C7—C8	120.5 (4)	H30A—C30—H30C	109.5
C6—C7—H7	119.7	H30B—C30—H30C	109.5
C8—C7—H7	119.7	H1W—O1W—H2W	105 (4)
C9—C8—C7	119.5 (4)		
			
C17—N1—C1—C2	86.2 (3)	C10—C11—C16—O2	7.2 (4)
C3—N1—C1—C2	−159.1 (3)	C12—C11—C16—C15	1.0 (4)
Dy1—N1—C1—C2	−35.6 (3)	C10—C11—C16—C15	−173.6 (3)
N1—C1—C2—N2	55.5 (4)	C1—N1—C17—C18	−79.8 (3)
C23—N2—C2—C1	74.6 (3)	C3—N1—C17—C18	163.9 (3)
C10—N2—C2—C1	−165.5 (3)	Dy1—N1—C17—C18	43.2 (3)
Dy1—N2—C2—C1	−43.9 (3)	C22—N3—C18—C19	2.1 (5)
C17—N1—C3—C4	177.2 (3)	Dy1—N3—C18—C19	179.2 (3)
C1—N1—C3—C4	58.2 (3)	C22—N3—C18—C17	−174.5 (3)
Dy1—N1—C3—C4	−64.4 (3)	Dy1—N3—C18—C17	2.6 (4)
N1—C3—C4—C9	61.3 (4)	N1—C17—C18—N3	−32.3 (4)
N1—C3—C4—C5	−122.4 (3)	N1—C17—C18—C19	151.0 (3)
C9—C4—C5—C6	−1.4 (5)	N3—C18—C19—C20	−3.4 (6)
C3—C4—C5—C6	−177.7 (4)	C17—C18—C19—C20	173.1 (4)
C4—C5—C6—C7	−0.3 (6)	C18—C19—C20—C21	1.8 (7)
C5—C6—C7—C8	0.9 (7)	C19—C20—C21—C22	0.9 (6)
C6—C7—C8—C9	0.2 (6)	C18—N3—C22—C21	0.8 (5)
Dy1—O1—C9—C8	126.2 (3)	Dy1—N3—C22—C21	−176.1 (3)
Dy1—O1—C9—C4	−54.6 (4)	C20—C21—C22—N3	−2.3 (6)
C7—C8—C9—O1	177.3 (3)	C10—N2—C23—C24	77.3 (3)
C7—C8—C9—C4	−1.9 (5)	C2—N2—C23—C24	−166.1 (3)
C5—C4—C9—O1	−176.8 (3)	Dy1—N2—C23—C24	−46.2 (3)
C3—C4—C9—O1	−0.4 (4)	C28—N4—C24—C25	1.4 (5)
C5—C4—C9—C8	2.4 (5)	Dy1—N4—C24—C25	178.0 (2)
C3—C4—C9—C8	178.9 (3)	C28—N4—C24—C23	179.0 (3)
C23—N2—C10—C11	−63.2 (3)	Dy1—N4—C24—C23	−4.4 (3)
C2—N2—C10—C11	176.9 (3)	N2—C23—C24—N4	36.1 (4)
Dy1—N2—C10—C11	57.1 (3)	N2—C23—C24—C25	−146.3 (3)
N2—C10—C11—C12	121.6 (3)	N4—C24—C25—C26	−1.3 (5)
N2—C10—C11—C16	−63.9 (4)	C23—C24—C25—C26	−178.8 (3)
C16—C11—C12—C13	−1.4 (5)	C24—C25—C26—C27	1.2 (6)
C10—C11—C12—C13	173.1 (3)	C25—C26—C27—C28	−1.2 (6)
C11—C12—C13—C14	0.4 (5)	C24—N4—C28—C27	−1.4 (5)
C12—C13—C14—C15	1.0 (6)	Dy1—N4—C28—C27	−177.8 (3)
C13—C14—C15—C16	−1.4 (6)	C26—C27—C28—N4	1.3 (6)
Dy1—O2—C16—C15	−129.4 (3)	Dy1^i^—O4—C29—O3	−174.1 (2)
Dy1—O2—C16—C11	49.8 (4)	Dy1^i^—O4—C29—C29^i^	6.2 (4)
C14—C15—C16—O2	179.6 (3)	Dy1—O3—C29—O4	−173.9 (2)
C14—C15—C16—C11	0.4 (5)	Dy1—O3—C29—C29^i^	5.8 (4)
C12—C11—C16—O2	−178.2 (3)		

Symmetry code: (i) −*x*+2, −*y*+1, −*z*+1.

### (µ-Oxalato)bis{[N,N'-bis(2-oxidobenzyl-κO)-N,N'-bis(pyridin-2-ylmethyl-κN)ethylenediamine-κ2N,N']dysprosium(III)}–methanol–water (1/4/4) (2) Hydrogen-bond geometry (Å, º)

**Table:**

*D*—H···*A*	*D*—H	H···*A*	*D*···*A*	*D*—H···*A*
O30—H30···O1	0.84	1.80	2.636 (4)	178
C1—H1*B*···O1*W*^ii^	0.99	2.58	3.448 (4)	146
O1*W*—H1*W*···O2^i^	0.82 (2)	1.97 (2)	2.785 (4)	167 (5)
O1*W*—H2*W*···O30	0.86 (2)	1.95 (3)	2.759 (5)	158 (5)

Symmetry codes: (i) −*x*+2, −*y*+1, −*z*+1; (ii) −*x*+1, −*y*+1, −*z*+1.
